# Genomic Surveillance of 4CMenB Vaccine Antigenic Variants among Disease-Causing *Neisseria meningitidis* Isolates, United Kingdom, 2010–2016

**DOI:** 10.3201/eid2404.171480

**Published:** 2018-04

**Authors:** Charlene M.C. Rodrigues, Jay Lucidarme, Ray Borrow, Andrew Smith, J. Claire Cameron, E. Richard Moxon, Martin C.J. Maiden

**Affiliations:** University of Oxford, Oxford, UK (C.M.C. Rodrigues, E.R. Moxon, M.C.J. Maiden);; Public Health England, Manchester, UK (J. Lucidarme, R. Borrow);; Glasgow Royal Infirmary, Glasgow, Scotland, UK (A. Smith); University of Glasgow, Glasgow (A. Smith);; Health Protection Scotland, Glasgow (J.C. Cameron)

**Keywords:** Neisseria meningitidis, bacteria, meningococci, genomic surveillance, molecular epidemiology, multilocus sequence typing, MLST, vaccines, meningococcal vaccines, 4CMenB, Bexsero, vaccine antigenic variants, meningitis/encephalitis, invasive meningococcal disease, IMD, whole-genome sequencing, epidemiologic year, United Kingdom

## Abstract

In September 2015, 4CMenB meningococcal vaccine was introduced into the United Kingdom infant immunization program without phase 3 trial information. Understanding the effect of this program requires enhanced surveillance of invasive meningococcal disease (IMD) *Neisseria meningitidis* isolates and comparison with prevaccination isolates. Bexsero Antigen Sequence Types (BASTs) were used to analyze whole-genome sequences of 3,073 prevaccine IMD *N. meningitidis* isolates obtained during 2010−2016. Isolates exhibited 803 BASTs among 31 clonal complexes. Frequencies of antigen peptide variants were factor H binding protein 1, 13.4%; Neisserial heparin-binding antigen 2, 13.8%; *Neisseria* adhesin A 8, 0.8%; and Porin A-VR2:P1.4,10.9%. In 2015−16, serogroup B isolates showed the highest proportion (35.7%) of exact matches to >1 Bexsero components. Serogroup W isolates showed the highest proportion (93.9%) of putatively cross-reactive variants of Bexsero antigens. Results highlighted the likely role of cross-reactive antigens. BAST surveillance of meningococcal whole-genome sequence data is rapid, scalable, and portable and enables international comparisons of isolates.

*Neisseria meningitidis* is an accidental human pathogen that is carried asymptomatically in the nasopharynx of 1%–40% of the population, depending on age and social behavior (*1*,*2*). In England and Wales, invasive meningococcal disease (IMD), comprising septicemia, meningitis, or both, develops in ≈2 persons/100,000 population/year (*3*). Patients have nonspecific symptoms early in their illness, but their conditions can deteriorate rapidly, with case-fatality rates of 5%–17% and physical and psychological sequelae in one third of surviviors (*3*–*5*). Consequently, vaccination represents the optimal strategy for IMD prevention.

Meningococci are commonly characterized according to their expressed capsular polysaccharides, which define serogroups; 6 serogroups (A, B, C, W, X, and Y) cause most cases of IMD. The capsular antigens are major virulence factors, and efficacious A, C, W, and Y polysaccharide−based vaccines are used worldwide. However, serogroup B capsular polysaccharides are poorly immunogenic and share structural similarity to carbohydrates found in human tissues (*6*). The first serogroup B vaccines were derived from outer membrane vesicles (OMVs) for use in epidemics caused by single strains defined by genotype and Porin A (PorA) type (*7*). Genotype or clonal complex (CC), identified by multilocus sequence typing (MLST), groups related organisms and is useful for categorizing IMD phenotype, antimicrobial drug resistance, and vaccine antigens (*8*,*9*).

The United Kingdom and Ireland are among high-income countries with the highest incidence of IMD (*10*). The United Kingdom has low-incidence endemic disease and periods of hyperendemicity, which changes with frequency of hyperinvasive bacterial genotypes (*10*). Historically, endemic serogroup B IMD predominated and was caused by multiple CCs, especially hyperinvasive lineages CC41/44, CC269, CC213, and CC32 (*10*). In the 1990s, hyperendemic serogroup C IMD caused by cc11 (C:CC11) prompted introduction of infant meningococcal C conjugate vaccination and a catch-up campaign, which reduced disease incidence and carriage of C:CC11 (*11*). The United Kingdom experienced another period of hyperendemicity starting in 2012 with increasing incidence of W:CC11, lineage 11.1 meningococci, first identified in South America (*12*).

Because of diversity of CCs responsible for IMD, OMVs derived from single CCs do not offer sufficiently broad protection. Alternative vaccine candidates have been developed. These candidates are composed of subcapsular proteins prevalent among many *N. meningitidis* strains. However, their protective potential has been complicated by meningococcal diversity (*13*–*15*). Two vaccines were licensed in 2013: 4CMenB (Bexsero; GlaxoSmithKline, Brentford, UK) for infants in Europe and bivalent recombinant lipoprotein rLP2086 (Trumenba; Pfizer, New York, NY, USA) for persons 10–25 years of age in the United States (*16*–*18*). The 4CMenB vaccine contains multiple proteins: factor H binding protein (fHbp); Neisserial heparin-binding antigen (NHBA); *Neisseria* adhesin A (NadA); and PorA P1.7–2,4 from the New Zealand OMV vaccine (MeNZB) (*16*). The rLP2086 vaccine contains 2 fHbp variants, 1 each from subfamily A (A05) and subfamily B (B01) (*17*).

Bexsero was implemented into the United Kingdom immunization schedule on September 1, 2015, as a 2-dose priming course for infants at 2 and 4 months of age and a booster at 12 months of age, intended as an efficacious strategy for those at highest risk within the constraints of cost-effectiveness (*18*). As with previously licensed meningococcal vaccines, efficacy studies were precluded because of the rarity of IMD, and the Meningococcal Antigen Typing System (MATS) was developed, which estimated 73% (95% CI 57%–87%) vaccine coverage for UK isolates (*19*,*20*).

An appreciation of meningococcal antigenic diversity and persistence over time is required to determine the degree and longevity of coverage provided by protein vaccines. Real-time, continuous, and high-throughput methods are needed to identify characteristics of circulating meningococci on a national scale, especially the frequency distribution of vaccine antigens. This characterization can be performed rapidly and reproducibly by using whole-genome sequencing (WGS) of IMD *N. meningitidis* isolates, for which data are publicly available online in the PubMLST database (https://pubmlst.org/neisseria) (*21*).

A novel nomenclature, Bexsero Antigen Sequence Types (BASTs), was devised to describe Bexsero antigenic variants (*22*). There were strong, nonoverlapping associations between BAST and CC, with an estimated 58.3%–60.3% Bexsero coverage including the antigenic variants in Bexsero or cross-reactive variants (*22*). We cataloged genomic diversity of Bexsero vaccine antigens by using web-accessible platforms incorporating BAST. This study provides a reference point for changes in population structure of IMD-causing meningococci in the United Kingdom before introduction of Bexsero.

## Materials and Methods

A total of 3,073 meningococci were isolated from culture-confirmed IMD cases in the United Kingdom during epidemiologic years (July 1−June 30) 2010–2016. For the purposes of this study, the prevaccine period includes 2015−16 because implementation of Bexsero started on September 1, 2015, and many infants were not fully vaccinated during the peak IMD season (December 2015−February 2016). The 3,073 isolates represented ≈55% of laboratory-confirmed cases of IMD (Table 1) because recovery of isolates reflects differential survival in artificial media, susceptibility to antimicrobial drugs given before venipuncture, or small-volume pediatric blood cultures.

**Table 1:** Geographic distribution of culture-confirmed invasive meningococcal disease isolates that have undergone whole- genome sequencing, United Kingdom, 2010–2016*

Year	No. isolates by region†		No. laboratory-confirmed cases (% isolates)		Serogroup, no. isolates‡

1	2	3	4	Total	PHE	PHS	A	B	C	E	NG	W	W/Y	X	Y	Z
2010−11	464	37	36	13	550		1,009 (46.0)	76 (47.4)		1	423	13	1	5	27	2	1	76	1
2011−12	370	28	30	11	439		730 (50.7)	56 (53.6)		0	311	18	1	4	32	1	0	72	0
2012−13	415	29	34	13	491		769 (54.0)	66 (51.5)		0	325	29	0	7	51	1	0	78	0
2013−14	367	27	30	12	436		636 (57.7)	60 (50.0)		0	240	24	0	1	92	2	0	77	0
2014−15	483	19	55	15	572		724 (66.7)	65 (84.6)		0	283	23	0	5	172	2	0	87	0
2015−16	488	23	62	12	585		805 (60.6)	99 (62.6)		0	238	34	2	8	197	2	0	104	0
Total	2,587	163	247	76	3,073		4,673 (55.4)	442 (55.9)		1	1,820	141	4	30	571	10	1	494	1
*Laboratory-confirmed cases included culture-confirmed and PCR-confirmed cases. Data included in this study represented 55.4% of all laboratory-confirmed cases in England and 55.9% of those in Scotland. NG, nongroupable; PHE, Public Health England; PHS, Public Health Scotland. †1, England; 2, Wales; 3, Scotland; 4, Northern Ireland. ‡W/Y serogroups were combined because of inconclusive serogrouping results.																			

### Genomic Analysis

WGS was part of the Meningitis Research Foundation Meningococcus Genome Library initiative (*10*). Genomes were assembled by using Velvet and VelvetOptimiser, uploaded to the PubMLST database, and annotated by using *Neisseria* Sequence Typing Database numbers (NEIS) for all loci. Analysis was undertaken by using the gene-by-gene approach with the Bacterial Isolate Genome Sequence Database to determine sequence type (ST), CC, and strain designation (*21*,*23*). Each isolate had associated provenance and phenotype data, including year, serogroup, and region.

We assigned BASTs as described (*22*). Nucleotide sequences of *fhbp*, *nhba*, *nadA*, and *porA* variable regions 1 and 2 (VR1 and VR2) were translated to deduce peptide sequences, and variant numbers were assigned by using established nomenclatures (*24*–*26*). Unique combinations of the 5 components were assigned a BAST number in order of discovery; BAST-1 corresponds to the vaccine constituents: fHbp 1, NHBA 2, NadA 8, and PorA 7–2,4 (*22*). Data were manually curated to confirm the absence of *fhbp*, *nhba*, *nadA*, and *porA*, and isolates were assigned peptide designation 0 (null). If nucleotide sequences contained a frameshift mutation, peptide designation 0 (null) was assigned. Peptide variants were not assigned if the complete gene was not available because of sequencing or assembly issues (*22*,*27*).

For assessment of Bexsero antigenic variants expected to be prevented by vaccination (coverage), we compared the genotypic profile (BAST) of isolates with those of vaccine antigenic variants. The term exact match indicates isolates having >1 of 4 Bexsero antigenic variants (fHbp 1, NHBA 2, NadA 8, and PorA-VR2:4). The term cross-reactive match indicates isolates having >1 variant that can potentially be recognized by Bexsero-induced antibodies, demonstrating a possible cross-protective immune response in humans. 

These variants were previously identified by using MATS analysis, which at the time of writing, was the most extensively used method for assessing qualitative and quantitive differences in antigens (*20*,*28*,*29*). Variants were considered putatively cross-reactive; fHbp peptides 4, 13, 14, 15, 37, 232 and NadA variants 1 or 2/3. NadA peptides were included because of potential discrepancies between in vitro and in vivo NadA expression (*20*,*30*). Cross-reactive NHBA peptides were not included because of lack of data on the breadth of peptides covered by Bexsero. Genomic analysis has not been used to infer protein expression or immunologic cross-reactivity per se.

We performed all statistical analyses by using R software version 3.2.4 (https://www.r-project.org/). We calculated the Simpson index of diversity by using the Vegan package in R software to assess diversity of BAST; values closer to 1 indicated greater diversity.

### Nomenclature of Antigenic Variants

There are 3 systems for classifying fHbp variants. The first system described 3 variants (1–3) on the basis of sequence similarity and cross-reactivity in serum bactericidal antibody (SBA) assays (*31*). The second system described 2 subfamilies, A and B (*32*). The third system, used in our study as part of the BAST scheme, assigned arbitrary numerical integers to unique deduced peptide sequences independent of variant/subfamily (*24*). NHBA peptide variants were assigned arbitrarily to unique peptide sequences. Updated nomenclature for NadA described 4 variants on the basis of peptide sequence homology: NadA-1, NadA-2/3, NadA-4/5, and NadA-6 (*26*). PorA nomenclature was based on nucleotide and peptide sequence homology and recognized the previous serologic classification: P1, followed by VR1 family-variant, and VR2 family-variant (e.g., P1.7–2,4) (*25*). All nomenclature is available in the PubMLST database.

## Results

### Distribution of Bexsero Vaccine Antigens

**Figure 1:**
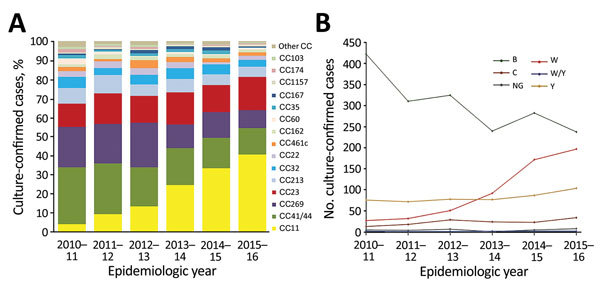
Clonal complex and serogroup distribution of invasive meningococcal disease isolates, United Kingdom, 2010–2016. A) Proportional contribution of each cc of disease-causing culture-confirmed meningococcal isolates by epidemiologic year. Other cc indicates ccs that were found in <20 isolates during the 6-year study period. B) Distribution of isolate serogroups by epidemiologic year. Serogroups shown had >10 isolates during the 6-year study period. Serogroups with <10 isolates (A, E, X, and Z) are shown in Table 1. CC, clonal complex; NG, nongroupable, W/Y, serogroups combined because of inconclusive serogrouping results.

For 2,922 isolates (95.1%), MLST, which was deduced from WGS data, identifed 645 STs with 2,866 (93.3%) isolates assigned to 1 of 31 CCs. We found variation in CC distribution over the 6-year period, with a 10-fold increase in cc11 and decreases in CC41/44 (172 to 56, 50.0%) and CC269 (108 to 52, 51.8%), which accounted for most serogroup B isolates (Figure 1).

### fHbp Peptide

**Figure 2:**
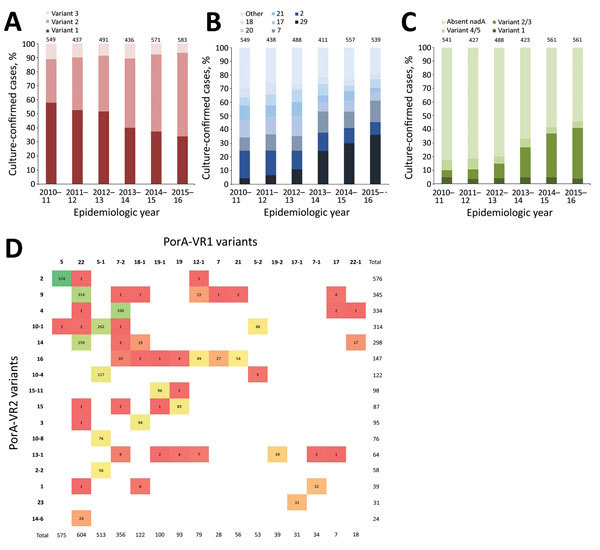
Distribution of 4CMenB vaccine antigenic variants among invasive meningococcal disease isolates, United Kingdom, 2010–2016. A) Proportion of isolates with fHbp variants 1, 2, and 3 by epidemiologic year. Peptide 1 is found in the Bexsero 4CMenB vaccine (GlaxoSmithKline, Bentford, UK), and cross-reactive variants included in this analysis are all variant 1 peptides. B) Proportion of isolates with the 7 most prevalent NHBA peptides by epidemiologic year; all other peptide variants are in “other.” Peptide 2 is contained in Bexsero. C) Proportion of isolates with NadA variants 1, 2/3, and 4/5 by epidemiologic year; there were no isolates with NadA variant 6. Peptide 8 (variant 2/3) is contained in Bexsero. Values above columns indicate number of unique peptides. D) Frequency distribution of PorA-VR1 (horizontal axis) and PorA-VR2 (vertical axis) variants. Variants shown were those that had >20 isolates in the collection from the United Kingdom during 2010–2016. Bexsero contains the MeNZB OMV vaccine components, including variants PorA P1.7–2,4. Color scales show the frequency of isolates from highest (green) to lowest (red). fHbp, factor H binding protein; NadA, *Neisseria* adhesin A; NHBA, Neisserial heparin-binding antigen; OMV, outer membrane vesicles; PorA, porin A.

An *fhbp* gene was present in 3,065 (99.7%) isolates, absent in 5 (1.6%), and not assigned in 3 (0.1%) because of incomplete sequence assembly. Across all serogroups, variant 2 fHbp peptides increased over the 6-year period, but most markedly in 2014−15 (313/572, 54.7%) and 2015−16 (347/585, 59.3%). From 2010−11 through 2012−13, variant 1 peptides predominated; from 2013−14 onwards, variant 2 peptides were more frequent than variant 1 peptides (Figure 2, panel A). Overall, there were 207 unique fHbp peptides in the collection: 109 variant 1, 43 variant 2, 54 variant 3, and 1 between variants 2 and 3.

Variant 1 peptides were present in 1,389 (45.3%) of 3,065 isolates, and 5 peptides accounted for 1,144 (82.4%) of 1,389 isolates: 4 (399, 28.7%), 13 (329, 23.7%), 15 (184, 13.2%), 14 (127, 9.1%), and 1 (105, 7.6%). The contribution of these 5 peptide variants among IMD *N. meningitidis* isolates decreased from 278 (50.5%) of 550 isolates in 2010−11 to 154 (26.3%) of 585 isolates in 2015−16. Peptide 4 was predominantly associated with B:CC41/44, whereas peptides 13 and 15 were mainly associated with B:CC269. Peptide 1, the variant in Bexsero, was present in 105 isolates, predominantly B:CC32, and decreased in incidence from 25 (4.5%) of 550 isolates in 2010−11 to 13 (2.2%) of 585 isolates in 2015−16.

Variant 2 peptides were present in 1,404 (45.8%) of 3,065 isolates; 6 peptides (22, 25, 19, 16, 21, and 24) accounted for 1,323 (94.2%) of 1,404 isolates. Peptide 22 (predominantly W:CC11) increased 13-fold from 15 isolates in 2010−11 to 198 isolates in 2015−16. Peptide 25, associated with Y:CC23, was consistently present through the period.

Variant 3 peptides were present in 274 (8.9%) of 3,065 isolates. Peptides 45, 47, and 31 were the most frequently occurring (predominantly B:CC213 and B:CC461).

### NHBA Peptide

The *nhba* gene was present in 2,986 (97.2%) isolates; 87 isolates were not assigned an allelic variant because of incomplete sequence assembly. There were 163 unique NHBA peptides, and 4 isolates were assigned null (0), considered unlikely to express functional proteins because of frameshift mutations in the *nhba* coding region. The most frequently occurring peptide was 29 (568/2,986, 19.0%), which increased 7-fold during the study period and was associated with W:CC11. The second most common was peptide 2, the variant in Bexsero, which decreased in incidence from 111 (20.2%) of 550 isolates in 2010−11 to 50 (8.5%) of 585 isolates in 2015−16; most (411/424) isolates belonged B:CC41/44. Other prevalent NHBA variants were 7, 17, 21, 20, and 18, which, with 29 and 2, accounted for 2,258 (75.6%) of 2,986 isolates (Figure 2, panel B).

### NadA Peptide

We found 2,036 (66.3%) isolates in which *nadA* was absent, 34 (1.1%) with an insertion element, and 38 (1.2%) not assigned an allelic variant because of incomplete sequence assembly. A total of 2,128 (69.2%) isolates were assigned NadA peptide null (0) because of absence of *nadA* genes or presence of frameshift mutations or insertion elements disrupting the coding sequence. Of the prevalent CCs, NadA peptide was absent from all isolates belonging to CC41/44, CC23, CC22, CC162, CC35, CC167, CC103, and CC282. Of the 907 (29.5%) isolates with NadA peptides present, there were 135 (4.4%) NadA-1 variants, 592 (19.3%) NadA-2/3 variants, and 180 (5.9%) NadA-4/5 variants; there were no NadA-6 variants (Figure 2, panel C). The proportion of isolates with NadA peptides increased from 96 (17.5%) of 550 isolates in 2010−11 to 257 (43.9%) of 585 isolates in 2015−16. Among these isolates, there were 23 unique peptides. The most common, peptide 6 (NadA-2/3), increased from 14 (2.5%) of 550 isolates in 2010−11 to 187 (40.0%) of 585 isolates in 2015−16. Peptide 79 (NadA-4/5, contained a homopolymeric tract resulting in phase variation) was the second most common, designated as phase variable on, and occurred in 164 isolates, predominantly B:CC213. Peptide 8 (NadA-2/3), found in Bexsero, was present in 0.8% (26/3,073) of isolates (Y:CC174, B:269, B:CC60, B:CC18, A:CC5).

### Por A Peptide

The *porA* gene was present in 3,011 (98.0%) of 3,073 isolates. For 54 isolates, no nucleotide sequence allele was assigned because of incomplete sequence assembly. For 3,064 (99.7%) isolates, predicted VR1 and VR2 peptides were obtained. PorA-VR2 variant 4 was found in 336 (10.9%) isolates, and showed decreasing incidence from 90 (16.5%) of 547 isolates in 2010−11 to 38 (6.5%) of 585 isolates in 2015−16. PorA-VR2 variant 4 containing isolates were predominantly B:cc41/44 (302/336, 89.9%) but also B:CC162, B:CC269, B:CC213, B:CC32, and B:CC60 and were associated with PorA-VR1 variants 7–2 (n = 330), 17 (n = 2), 12–3 (n = 2), 22 (n = 1), and 22–1 (n = 1) (Figure 2, panel D).

### BAST

**Figure 3:**
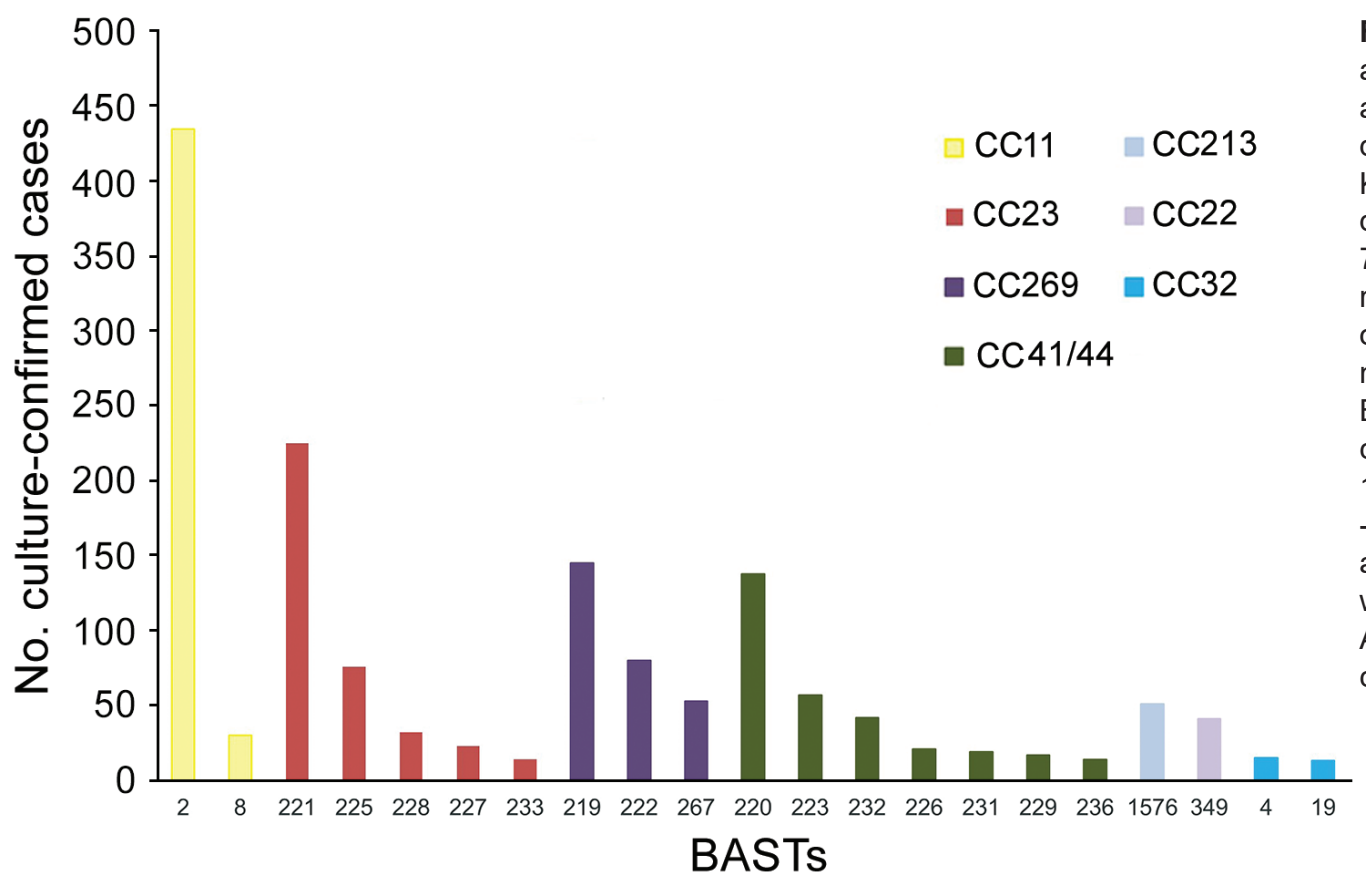
Nonoverlapping association of BAST and cc among invasive meningococcal disease isolates, United Kingdom, 2010–2016. Frequency distribution of BAST by CC for the 7 most frequently found ccs that represent 82.4% (2,533/3,073) of culture-confirmed invasive meningococcal disease isolates. BAST-220, -223, -4, and -19 contain an exact match with BAST-1. BAST-2, -8, -219, -222, -232, -226, -231, -229, and -236 contain a potentially cross-reactive match with BAST-1. BAST, Bexsero Antigen Sequence Type; CC, clonal complex.

**Figure 4:**
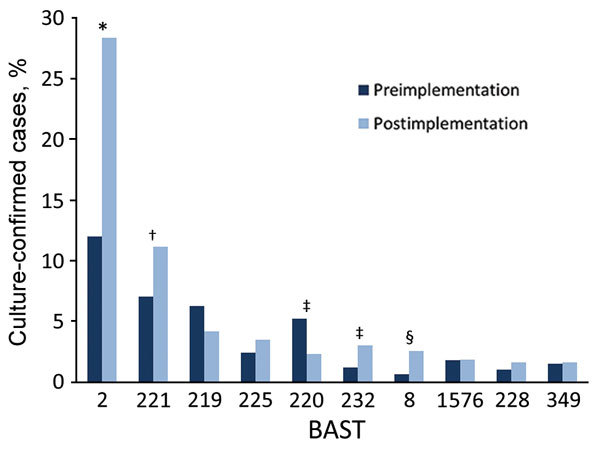
Changes in BAST prevalence before and after Bexsero implementation among invasive meningococcal disease isolates, United Kingdom, 2010–2016. Frequency of BASTs is shown for the period before implementation of Bexsero vaccine, July 2010–August 2015 (dark blue), and after implementation, September 2015–June 2016 (light blue). The most frequently occurring BASTs preimplementation were 2,¶ 221, 219,¶ 220,# 222,¶ 267, 225, 223,# 1576, and 349. The most frequent BASTs postimplementation were 2,¶ 221, 219,¶ 225, 220,# 232,¶ 8,¶ 1576, 228, and 349. *p<0.00001; †p<0.01; ‡p<0.05; §p<0001; ¶BAST contains a potentially cross-reactive match to BAST-1; #BAST contains an exact match to BAST-1. BASTs with significant changes preimplementation and postimplementation were BAST-2 (fHbp 22, NHBA 29, NadA 6, PorA-VR1:5, and PorA-VR2:2), p<0.00001; BAST-221 (25; 7; 0; 5–1; 10–1), p = 0.006; BAST-220 (4; 2; 0; 7–2; 4), p = 0.02; BAST-232 (4; 2; 0; 12–1; 16), p = 0.01; and BAST-8 (22; 29; 121; 5; 2), p = 0.0005. BAST, Bexsero Antigen Sequence Type.

We determined 803 unique BASTs for 2,917 (94.9%) isolates. The ratio of BASTs per isolate was calculated by dividing the number of unique BASTs by the number of isolates. This ratio decreased from 0.416 in 2010−11 to 0.265 in 2015−16. The Simpson index of diversity ranged from 0.976 in 2010−11 to 0.902 in 2015−16 (Table 2). We found strong, nonoverlapping association of BAST and cc (Figure 3). When we compared BAST prevalence preimplementation (cases during July 1, 2010−September 1, 2015) and postimplementation (cases during September 1, 2015−June 30, 2016), we found statistically significant increases in BAST-2 (cc11), BAST-221 (cc23), BAST-232 (cc41/44), and BAST-8 (cc11) and a decrease in BAST-220 (cc41/44) (Figure 4).

**Table 2:** Number of unique BASTs and measures of diversity among invasive meningococcal disease isolates, United Kingdom, 2010–2016*

Epidemiologic year	No. unique BASTs	No. isolates	No. BASTs/isolate	Simpson index of diversity
2010−11	229	550	0.416	0.976
2011–12	187	439	0.426	0.977
2012–13	226	491	0.460	0.976
2013–14	181	436	0.415	0.960
2014–15	199	572	0.348	0.924
2015–16	155	585	0.265	0.902
Total	803	3,073	0.261	0.963

*BASTs, Bexsero Antigen Sequence Types.

### BAST Distribution by Serogroup

Although Bexsero is licensed for serogroup B IMD, its components might be present on the surface of meningococci, independent of capsular type. Therefore, we analyzed distribution of Bexsero antigens by serogroup. Serogroup B isolates had the highest proportion of exact matches to >1 Bexsero antigen: 155 (36.6%) of 423 isolates in 2010−11 and 85 (35.7%) of 238 isolates in 2015−16, predominantly cc41/44. The proportion of isolates with exact or potentially cross-reactive antigens was 293 (69.3%) of 423 isolates in 2010−11 and 149 (62.6%) of 238 isolates in 2015−16 and represented cc269, cc41/44, cc32, cc213, cc60, cc1157, cc18, cc162, cc11, cc461, cc35, cc167, and cc254. The potentially cross-reactive variants included fHbp peptides 4, 13, 14, and 15 (956/1,144) and NadA peptide 1 (118/1,144).

For serogroup C, 8/141 isolates had >1 exact match to Bexsero components, but numbers varied each year depending on predominant cc. There were 2 (15.4%) of 13 matches to Bexsero components in 2010−11 and 3 (13.0%) of 23 matches to Bexsero components in 2014−15; isolates belonged to CC32. No isolates were exact matches in 2011−12 and 2015−16 (predominantly cc11). The proportion of isolates also having potentially cross-reactive antigens increased from 4 (30.8%) of 13 in 2010−11 to 25 (91.3%) of 34 in 2014−15. The antigenic variants were fHbp (50 isolates, predominantly peptide 13) and NadA (50 isolates, predominantly peptides 121, 127, and 1), largely reflecting secular changes in cc distribution with increases in CC11, CC269, CC32, and CC174.

Of 571 serogroup W isolates, 1 (0.2%) CC11 isolate was an exact match to Bexsero components (fHbp 1). When we included matches to potentially cross-reactive antigens, there were 14 (51.9%) of 27 isolates in 2010−11, which increased to 185 (93.9%) of 197 isolates in 2015−16. This increase was caused by NadA 6 in 500 (87.6%) of 571 isolates and fHbp 13 in 7 (1.2%) of 571 isolates, all of which belonged to lineage 11.1, W:cc11.

Serogroup Y disease isolates showed exact matches to Bexsero components in 7 (9.2%) of 76 isolates in 2010−11, which decreased to 3 (2.9%) of 104 isolates in 2015−16. When potential cross-reactive antigens were included, matches ranged from 9 (11.8%) of 76 isolates in 2010−11 to 3 (2.9%) of 104 isolates in 2015−16 and represented CC174, CC23, CC22, and CC11.

## Discussion

The requirement for vaccines to protect against serogroup B meningococci from multiple ccs led to development of multipeptide vaccines, such as 4CMenB (Bexsero) and bivalent rLP2086 (Trumenba) (*16*,*17*). Bexsero was introduced into the UK infant immunization schedule in September 2015, supported by data from the MATS assay that estimated 73% IMD *N. meningitidis* isolate coverage in England and Wales in 2007–08 (*20*).

We report a comprehensive evaluation of the frequency distribution of Bexsero antigen peptide variants in IMD *N. meningitidis* isolates, which used a national collection of WGS of culture-confirmed cases from 2010–2016. The frequency distribution of individual vaccine antigens was correlated with the distribution of meningococcal ccs in the United Kingdom over time. During 2010–2016, high diversity of 803 BASTs and 31 ccs emphasized the broad coverage required of peptide-based vaccines if they are to protect against endemic disease caused by multiple ccs. The most frequently occurring BASTs (20 representing 44.1% of serogroup B isolates) could provide useful information for future vaccine formulations.

Bexsero components correspond to BAST-1, fHbp 1, NHBA 2, NadA 8, PorA-VR1:7–2, and PorA-VR2:4. In this study, the incidence of individual BAST-1 antigens in serogroup B IMD cases in the United Kingdom during 2010–2016 was 5.4% (99/1,820) for fHbp, 23.0% (419/1,820) for NHBA, 0.3% (5/1,820) for NadA, and 18.4% (335/1,820) for PorA-VR2. Low levels of exact antigenic variants found in Bexsero imply that host immunogenicity to cross-reactive antigens would be necessary to provide the level of protection required by a national vaccination program, although this host response is also dependent on adequate protein expression, which cannot be determined solely from genomic analysis. In vitro studies comparing bactericidal killing of various fHbp variant 1 peptide−expressing meningococci (peptides 1, 2, 3, 4, 5, 10, 12, 13, 14, and 15) found cross-reactivity in postvaccination serum samples from adults, but functional activity in infants was limited to peptides 1 and 2 after immunization at 2, 4, and 6 months of age (*33*). 

Surface protein expression is also a major determinant of bactericidal killing. For fHbp, when heterologous bactericidal activity was tested, mouse antisera to peptide 1 produced positive titers against closely related peptide 4 regardless of expression level. For more distantly related peptides, such as peptide 15, higher protein expression was required (*34*).

At the time of writing, the most extensive estimation of cross-reactivity data for Bexsero had been collected by using the MATS assay. This assay quantifies expression and antigenic similarities of fHbp, NHBA, and NadA by sandwich ELISA and identifies PorA serosubtype by sequencing for each isolate (*20*,*35*). During development, the antigen measurements for fHbp, NHBA, and NadA were correlated to bactericidal killing with relative potency (RP) against 57 reference isolates tested by an SBA assay, determining likelihood of bacterial killing. Isolates with PorA-VR P1.4 peptide were considered to be covered, without further serologic testing (*35*). Contemporaneous MATS estimate of coverage for IMD *N. meningitidis* isolates from the United Kingdom during 2014–15 was 66% (95% CI 52%–80%) (*28*). However, the presence, cross-reactivity, and expression levels of antigenic variants in meningococci alone does not directly measure their susceptibility to bactericidal killing, which is also dependent on host innate and adaptive immune responses, a function not measured by the MATS assay. Therefore, this assay remains a surrogate for estimating functional activity against cross-reactive antigenic variants.

Among UK isolates we examined, the most frequent peptide variant 1 fHbp peptides were 4, 13, 15, 14, and 1. For most fHbp peptide 1 isolates tested by MATS, their RP lies above the positive bactericidal threshold (PBT), and these isolates are predicted to be killed by the pooled serum from Bexsero-vaccinated toddlers used in the assay (*20*). However, for other fHbp variant 1 peptides, there was marked variation in coverage estimates by MATS for isolates with the same peptide variant. Two MATS studies in Europe identified the RP for peptide 4 isolates to be most consistently above the PBT, but RP for peptide 13, 14, and 15 isolates spanned the PBT (*20*,*29*). The degree of protection afforded by Bexsero vaccination will be observed through postimplementation enhanced surveillance. With 2-dose vaccine uptake at 88.6%, early reports of vaccine efficacy were estimated to be 82.9% (95% CI 24.1%–95.2%) (*36*). If these high efficacy estimates, albeit with wide CIs, continue to show protection beyond that predicted by genotypic, phenotypic, or functional estimates, then synergistic activity or minor antigens might need to be considered, neither of which are quantified by MATS or BAST.

Coverage for nonserogroup B isolates by Bexsero-induced immunity was of special interest in the United Kingdom because of increasing IMD cases caused by W:CC11 from 2012, with severe and atypical IMD and high mortality rates (*37*). The principal change in this analysis was the increase of BAST-2 (22; 29; 6; 5; 2), a direct consequence of W:CC11 clonal expansion. Although conjugate ACWY vaccine was introduced in August 2015, it was targeted to teenagers, the age group with increased disease and highest risk for carriage (*38*). There is a paucity of supporting immunologic evidence for the role of Bexsero in protection against nonserogroup B isolates, but in a small case series of 6 W:CC11 isolates, all BAST-2, human SBA assay responses of >1:32 were observed by using pooled serum from infants vaccinated with 3 doses of Bexsero (*39*). Therefore, some protection might be provided to Bexsero-vaccinated infants and toddlers.

After implementation of Bexsero into the UK immunization schedule, long-term vaccine effectiveness will be established by enhanced IMD surveillance accompanied by characterization of meningococcal isolates (*36*). The methods used here are rapid, standardized, open-source, and readily applied to different settings (*22*,*40*). Use of WGS in the Meningitis Research Foundation Meningococcus Genome Library initiative in the United Kingdom to extract vaccine antigenic variant data enables rapid isolate characterization, surveillance of circulating meningococci, and monitoring of secular changes and the impact of all meningococcal vaccines in use (*10*,*22*). These data serve as a reference point against which effects of the national Bexsero program can be compared, and highlight reliance on cross-reactive variants to maintain effective protection. Finally, such data will be invaluable in development of novel vaccine formulations that ensure continued coverage.
